# Direct competition between DNA binding factors highlights the role of Krüppel-like Factor 1 in the erythroid/megakaryocyte switch

**DOI:** 10.1038/s41598-017-03289-5

**Published:** 2017-06-09

**Authors:** Laura J. Norton, Samantha Hallal, Elizabeth S. Stout, Alister P. W. Funnell, Richard C. M. Pearson, Merlin Crossley, Kate G. R. Quinlan

**Affiliations:** 1 School of Biotechnology and Biomolecular Sciences, University of New South Wales, Sydney, NSW 2052 Australia; 2 School of Molecular Bioscience, University of Sydney, Sydney, NSW 2006 Australia

## Abstract

The Krüppel-like factor (KLF) family of transcription factors play critical roles in haematopoiesis. KLF1, the founding member of the family, has been implicated in the control of both erythropoiesis and megakaryopoiesis. Here we describe a novel system using an artificial dominant negative isoform of KLF1 to investigate the role of KLF1 in the erythroid/megakaryocytic switch *in vivo*. We developed murine cell lines stably overexpressing a GST-KLF1 DNA binding domain fusion protein (GST-KLF1 DBD), as well as lines expressing GST only as a control. Interestingly, overexpression of GST-KLF1 DBD led to an overall reduction in erythroid features and an increase in megakaryocytic features indicative of a reduced function of endogenous KLF1. We simultaneously compared *in vivo* DNA occupancy of both endogenous KLF1 and GST-KLF1 DBD by ChIP qPCR. Here we found that GST-KLF1 DBD physically displaces endogenous KLF1 at a number of loci, providing novel *in vivo* evidence of direct competition between DNA binding proteins. These results highlight the role of KLF1 in the erythroid/megakaryocyte switch and suggest that direct competition between transcription factors with similar consensus sequences is an important mechanism in transcriptional regulation.

## Introduction

Erythrocytes and megakaryocytes are specialised cells of the haematopoietic system that are both derived from the megakaryocyte/erythroid progenitor (MEP). They have highly distinct gene expression profiles, functions and morphologies. Various transcription factors are involved in determining whether an MEP differentiates towards the erythroid or megakaryocyte lineage. These cells do, however, share a number of key transcription factors, the balance of which helps to determine which lineage an MEP will ultimately differentiate towards.

The Krüppel-like factor (KLF) family, consisting of 18 members to date^1–3^, has been widely implicated in haematopoiesis^3^. The family is characterised by three highly conserved C-terminal C2H2 zinc finger motifs that function as the DNA-binding domain^4^. These bind to CACCC-boxes and other GC rich elements, which are found at promoter and enhancer regions of their target genes^5–7^. Members of this family have been shown to function as both transcriptional activators and/or repressors, and have been divided into groups based on their functional similarities or their overall homology^2^.

The function of KLF1, the founding member of the family, has been extensively studied and its expression is largely restricted to erythroid cells^7^. KLF1 has been demonstrated to be a potent transcriptional activator, capable of binding CACCC-box motifs prevalent in the regulatory regions of many erythroid-associated genes^7–11^. It mediates its activation effects in part through interactions with CREB binding protein and p300 (CBP/p300)^12^. The most notable CACCC-box lies within the adult *β*-*globin* promoter, and KLF1 is central in driving the expression of this gene^8, 13^. It has been shown by chromatin immunoprecipitation (ChIP) to bind directly to the *β*-*globin* promoter *in vivo* ^14, 15^, and *Klf1* null mice die *in utero* at around E15 of severe *β*-*globin* deficiency and anaemia^16, 17^. Many other erythroid genes are also deregulated in the absence of *Klf1* ^10, 14, 18, 19^, including another member of the KLF family, the transcriptional repressor, *Krüppel*-*like factor 3* (*Klf3*)^20^. Expression of *Klf3* has since been demonstrated to be directly activated by KLF1^21^.

KLF1 has not only been shown to be important in promoting erythropoiesis, but has been identified as playing a role in repressing megakaryopoiesis. While KLF1 is highly upregulated as MEPs differentiate towards erythroid cells, it is conversely downregulated during megakaryocyte differentiation^22^. It has been shown that overexpression of KLF1 inhibits megakaryopoiesis^22^, and that loss of KLF1 promotes the expansion of megakaryocytes^22, 23^. In the absence of KLF1, murine erythroleukaemia (MEL) cells have been shown to upregulate the expression of megakaryocyte and platelet specific genes, such as *GpIIIa* and *GpIX*. It is believed that KLF1 is able to modulate its effect on megakaryopoiesis through the repression of the important megakaryocytic transcriptional regulator, *Fli1* ^22, 24, 25^.

Here we report a novel system using a GST tag fused upstream of the DNA binding domain of KLF1 (GST-KLF1 DBD) to investigate the roles of KLF1 in the erythroid/megakaryocytic switch. For these experiments, we selected the mouse erythroleukemia cell line, MEL cells. We developed MEL cell lines overexpressing GST-KLF1 DBD, as well as lines expressing GST alone as a negative control. Interestingly, these cells displayed a strong phenotypic effect, where cells expressing GST-KLF1 DBD displayed an overall reduced erythroid and increased megakaryocyte phenotype. This is consistent with the previously reported role of KLF1 in these processes and suggests that GST-KLF1 DBD has a dominant negative effect on endogenous KLF1. In order examine this dominant negative mechanism in greater detail, we investigated the *in vivo* binding of endogenous KLF1, and the binding of the ectopically expressed GST-KLF1 DBD. We found that GST-KLF1 DBD is able to out-compete endogenous KLF1 at a subset of target sites. Our results provide interesting new evidence to support a role for KLF1 in the erythroid/megakaryocyte switch, as well as indicating that transcription factor competition is an important regulation mechanism in eukaryotes *in vivo*.

## Results

### GST only and GST-KLF1 DBD constructs are detectably expressed in MEL cells, and GST-KLF1 DBD is capable of binding to DNA

**Figure 1:**
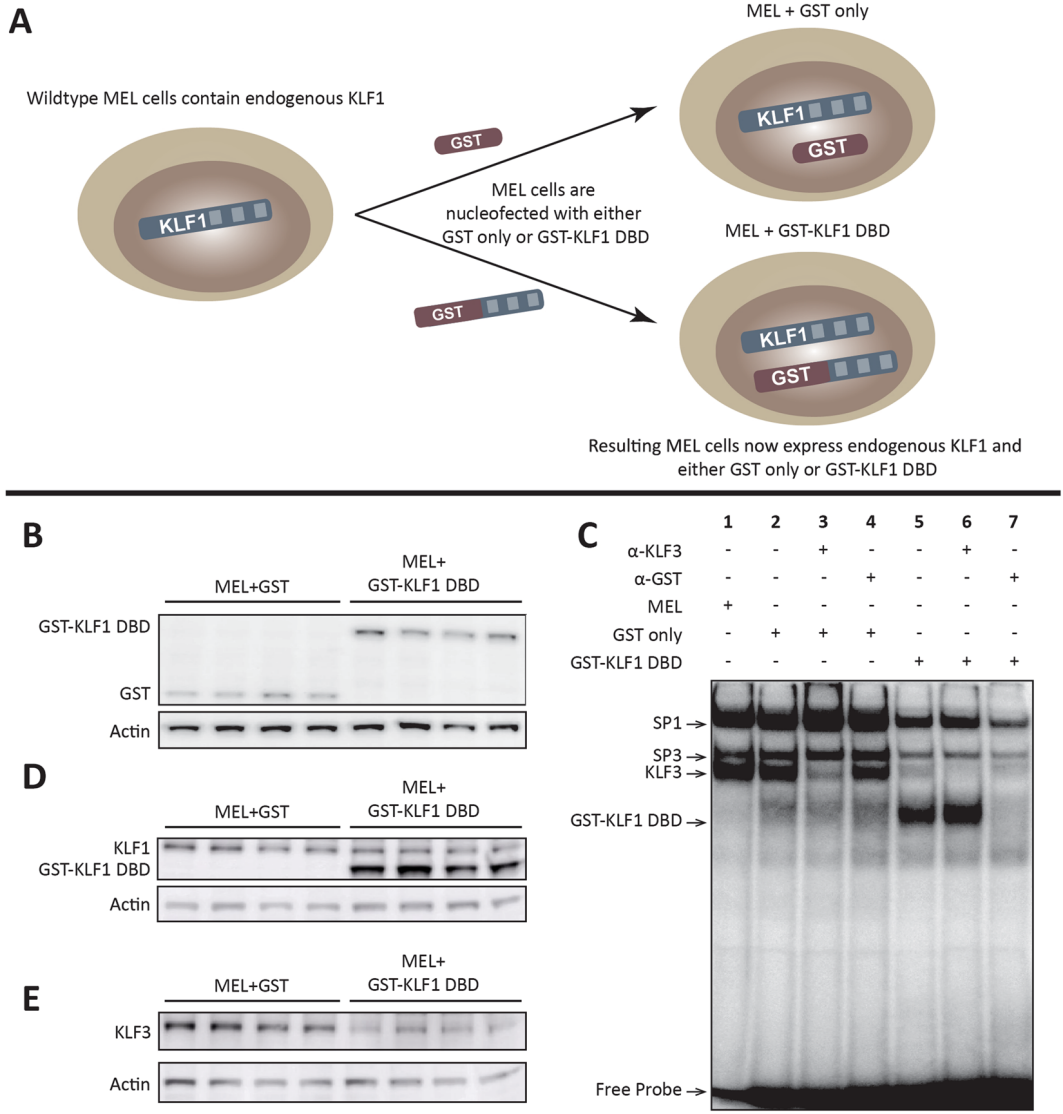
The GST-KLF1 DBD construct is capable of binding to the canonical KLF1 binding site and has a biological effect in MELs. (**A**) Schematic demonstrating the experimental design of the two different constructs nucleofected into MEL cells. (**B**) Western blots from MEL lines showing protein expression of GST and GST-KLF1 DBD for each clone. Actin is presented as a loading control. (**C**) EMSAs from representative MEL clones indicating GST-KLF1 DBD is capable of binding the canonical KLF1 consensus sequence (*β*-*globin* CACCC probe) *in vitro*. (**D**) Western blots from MEL clones with an antibody that recognises both endogenous KLF1 and GST-KLF1 DBD. β-Actin is presented as a loading control. (**E**) Western blots from MEL clones with an antibody that recognises KLF3, indicating reduced expression of endogenous KLF3 in cells overexpressing GST-KLF1 DBD compared to GST only clones. β-Actin is presented as a loading control. n = 4 for each construct.

We generated mouse erythroleukemia (MEL) cell lines stably expressing GST fused upstream of the first zinc finger, and thus encompassing the three zinc finger DNA binding domain, of KLF1 (261–376) (GST-KLF1 DBD). We also developed lines expressing GST only which served as negative controls (Fig. 1A). Four different clones from each line were selected as replicates, based on comparable levels of protein expression, as assessed by Western blot (Fig. 1B).

Electrophoretic mobility shift assays (EMSAs) were used to determine whether GST-KLF1 DBD is able to bind to the same sequence as KLF1 *in vitro*. We used the canonical KLF1 binding CACCC sequence, from the *β*-*globin* promoter^20^, and performed EMSAs in the MEL lines stably expressing GST alone and those expressing the GST-KLF1 DBD protein. This result demonstrates that the GST protein alone is unable to bind to DNA (Fig. 1C lanes 2–4), while the GST-KLF1 DBD protein is capable of binding the same consensus sequence as KLF1 *in vitro* (Fig. 1C, lanes 5–7).

We next sought to determine whether expression of GST-KLF1 DBD affected the endogenous levels of KLF1. Using an antibody that recognises both endogenous KLF1 and GST-KLF1 DBD in a Western blot with nuclear extracts from MEL cells, we show that GST-KLF1 DBD overexpression does not effect expression of endogenous KLF1 (Fig. 1D, quantitation in Fig. S1A). There was also no difference in the expression of *Klf1* at the mRNA level as determined by real time quantitative RT-PCR (qPCR) using primers that recognise endogenous KLF1 but not GST-KLF1 DBD (Fig. S1B). The level of GST-KLF1 DBD was 2–3 times that of the endogenous level of KLF1 protein in MEL cells expressing GST or GST-KLF1 DBD (Fig. 1D, quantitation in Fig. S1A).

Endogenous KLF3, which also binds to the CACCC sequence from the *β*-*globin* promoter, can be visualised on the EMSA. The identity of the band marked as KLF3 was confirmed by supershift with an antibody to KLF3 (Fig. 1C, lane 3). It was noted that the expression of KLF3 appeared reduced in cells overexpressing GST-KLF1 DBD (Fig. 1C lanes 5–7). In order to further investigate this finding in all four clones, we performed Western blots, examining the levels of KLF3. We found that KLF3 is indeed downregulated in MEL cells overexpressing the GST-KLF1 DBD construct compared to GST only cells (Fig. 1E). Densitometry analyses on both the EMSAs and the Western blots was performed to confirm these observations (Fig. S2A,B). We also investigated the levels of *Klf3* by qPCR and found it to be significantly reduced in MEL cells overexpressing GST-KLF1 DBD compared to GST only expressing cells (Fig. S2C). These data all confirm a reduction in the levels of KLF3 in cells expressing the GST-KLF1 DBD construct. To exclude the possibility that GST alone influences KLF3 levels, we remade new MEL cell clones stably overexpressing GST and GST-KLF1 DBD and, at the same time, generated control clonal MEL cells (grown in media without puromycin). The reduction in KLF3 protein and *Klf3* mRNA in GST-KLF1 DBD expressing MEL cells was replicated in these independent clones and GST alone had no effect on KLF3 expression when compared to clonal populations of the parental MEL cell line (Fig. S3). As *Klf3* expression is known to be activated by KLF1, the reduced *Klf3* expression in the cells expressing GST-KLF1 DBD suggests that this construct has a dominant negative effect on KLF1 activity.

### MEL cells expressing GST-KLF1 DBD show a reduced erythroid phenotype

**Figure 2:**
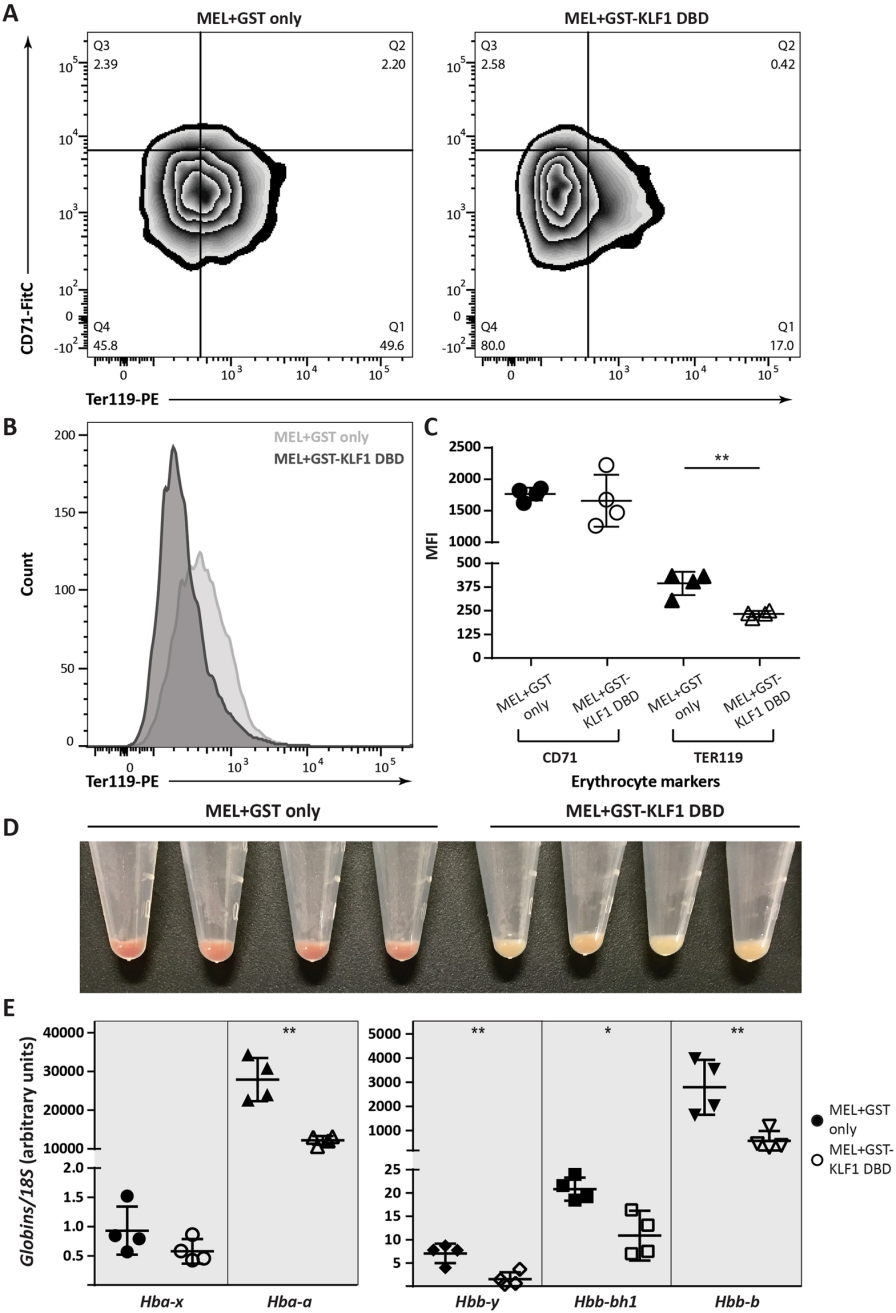
MEL cells overexpressing GST-KLF1 DBD display a less pronounced erythrocyte phenotype. (**A**) Representative flow cytometry plots of MEL + GST and MEL + GST-KLF1 DBD cells stained with antibodies against CD71 and TER119. (**B**) Histogram of flow cytometry for TER119 in a representative GST and GST-KLF1 DBD clone. (**C**) Statistical analysis of flow cytometry data for erythrocyte markers from all clones (**D**) MEL clones induced with DMSO. (**E**) Absolute levels of globin expression were analysed by qPCR in MEL + GST expressing clones compared to MEL + GST-KLF1 DBD expressing clones induced with DMSO. Expression levels were normalised to *18S* rRNA. n = 4 for each construct. Error bars shown represent standard error of the mean, p values indicate the difference between the means, *p < 0.05, **p < 0.01 (paired Student’s two-tailed *t* test).

We initially investigated the phenotype of the MEL lines by flow cytometry. Here, we found that the cells expressing GST-KLF1 DBD express markedly less TER119, the marker for mature erythroid cells, on their cell surface compared to cells expressing GST only (Fig. 2A–C). In contrast, the expression of the progenitor marker CD71 is not altered on the surface of these cells. We attempted to terminally induce these cells using DMSO as previously described^26^ to determine if this phenotype becomes more pronounced upon erythroid differentiation. Here we found that induction was blocked in the lines overexpressing GST-KLF1 DBD, as evidenced by their overall pale color compared to the hemoglobinized hue of the induced MEL cells expressing GST (Fig. 2D). Indeed, this reduction in globin expression in the GST-KLF1 DBD cells was also seen at the transcript level by qPCR, as the levels of the *α*-*globin* genes, *Hba*-*x* and *Hba*-*a*, and the *β*-*globin* genes, *Hbb*-*y*, *Hbb*-*bh1* and *Hbb*-*b*, are markedly reduced in these cells (Fig. 2E).

### MEL cells expressing GST-KLF1 DBD show an increased megakaryocyte phenotype

**Figure 3:**
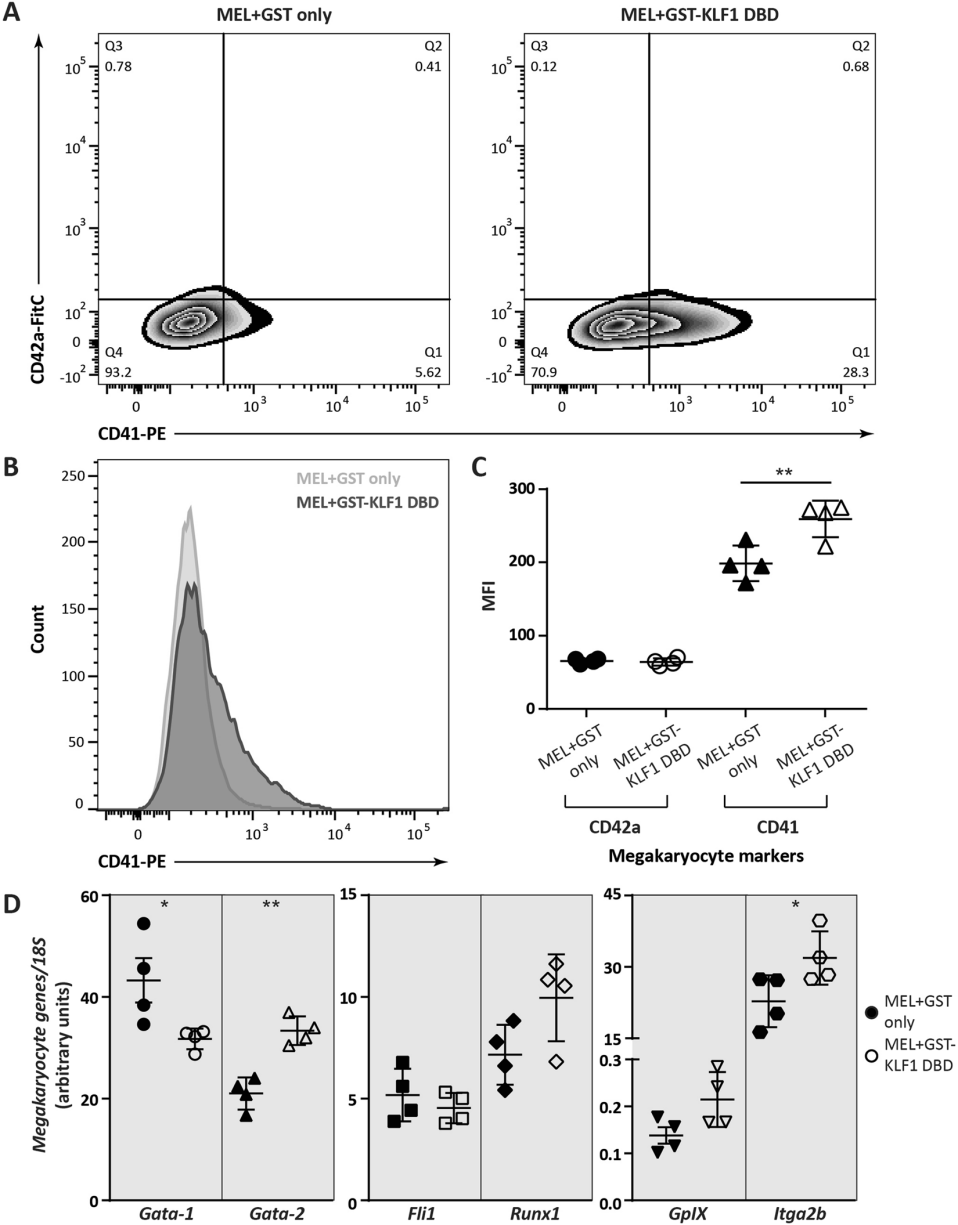
MEL cells overexpressing GST-KLF1 DBD display a more pronounced megakaryocyte phenotype. (**A**) Representative flow cytometry plots of MEL + GST and MEL + GST-KLF1 DBD cells stained with antibodies against CD42a and CD41. (**B**) Histogram of flow cytometry for CD41 in a representative GST and GST-KLF1 DBD clone. (**C**) Statistical analysis of flow cytometry data for erythrocyte markers from all clones. (**D**) Absolute levels of megakaryocyte related gene expression were analysed by real time qPCR in un-induced GST expressing clones compared to GST-KLF1 DBD expressing clones. Expression levels were normalised to *18S* rRNA. n = 4 for each construct. Error bars shown represent standard error of the mean, p values indicate the difference between the means, *p < 0.05, **p < 0.01 (paired Student’s two-tailed *t* test).

Considering KLF1’s role in establishing erythroid lineage commitment at the expense of megakaryopoiesis, we next sought to determine whether MEL cells expressing GST-KLF1 DBD display any increased megakaryocytic characteristics. To determine this, we investigated the megakaryocyte cell surface markers CD41 and CD42a by flow cytometry. We found that cells expressing GST-KLF1 DBD do indeed show increased levels of the megakayocytic marker CD41 on their surface compared to GST control cells (Fig. 3A–C). The marker CD42a, however, was unchanged across the two groups. We also examined the expression of megakaryocyte genes at the transcript level by qPCR, and found that the levels of *Gata2* and *Itga2b* were significantly increased in these cells, an increase in *Runx1* and *GpIX* appraoching significance (p = 0.07 and p = 0.06 respectively) was also obersved. *Gata1* levels were significantly decreased and interestingly, levels of *Fli1* were unchanged (Fig. 3D).

### GST-KLF1 DBD has a dominant negative effect on endogenous KLF1 at selective target sites

**Figure 4:**
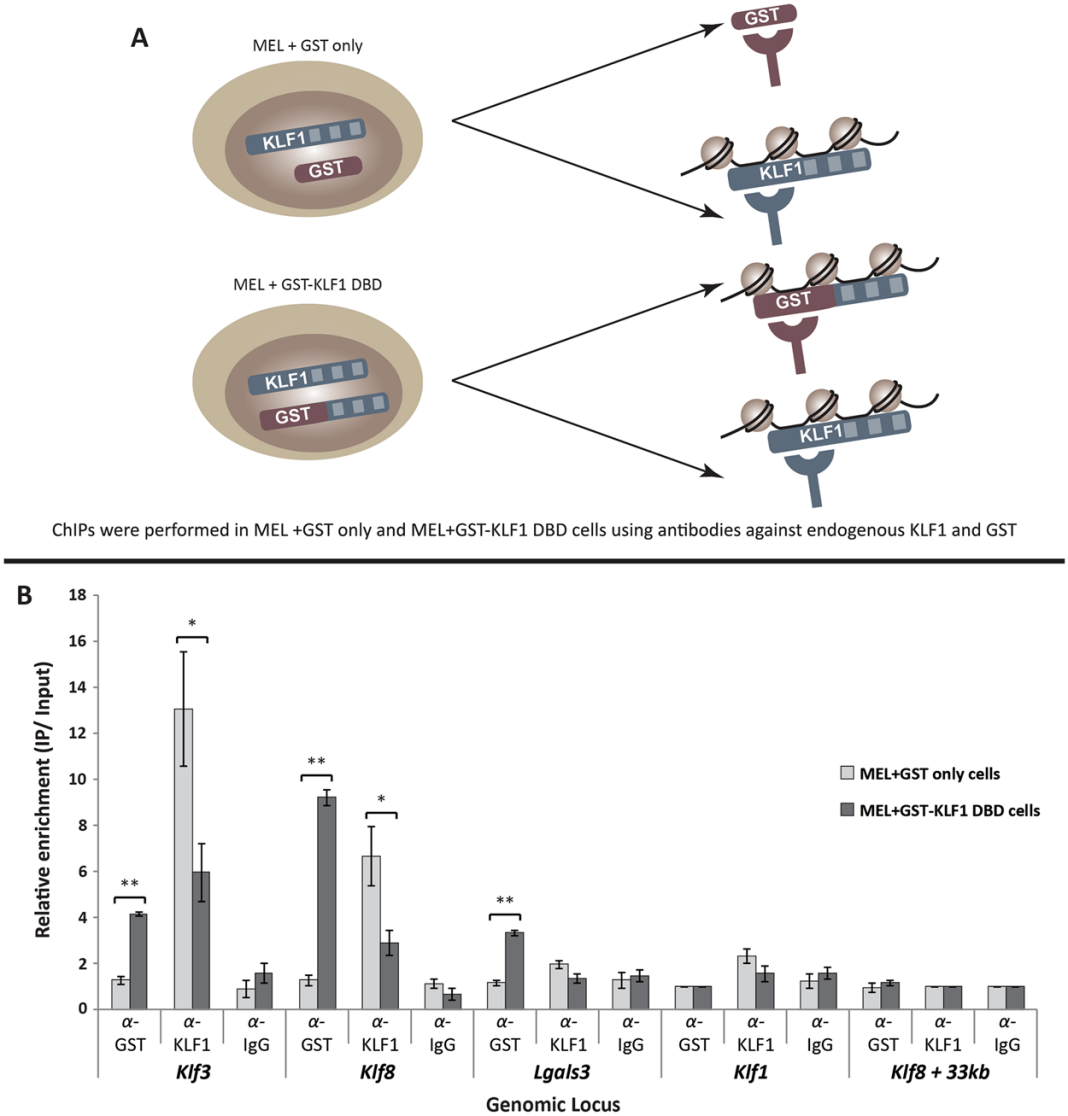
The GST-KLF1 DBD protein displaces endogenous KLF1 at a number of KLF1 target genes *in vivo*. (**A**) Schematic depicting the experimental design of the ChIP experiment. (**B**) ChIPs were performed using α-KLF1, α-GST and α-IgG in both in MEL + GST and MEL + GST-KLF1 DBD clones, (n = 4 for each group per IP). Data are represented as the fold-change enrichment of IP over input, where the negative control for each IP, the *Klf1* promoter for GST, and the *Klf8* +33 kb locus for both KLF1 and IgG, was set to 1. The *Klf3 1b* and *Klf8* promoters have been included as positive controls while the *Klf1* promoter, and *Klf3* +10 kb and *Klf8* +33 kb genomic regions are shown as negative controls. Error bars shown represent standard error of the mean, p values indicate the difference between the means, *p < 0.05, **p < 0.01 (paired Student’s two-tailed *t* test).

The results reported above are consistent with a dominant negative role of GST-KLF1 DBD on endogenous KLF1 in these cells. We hypothesised that the mechanism underlying this dominant negative effect is direct competition of GST-KLF1 DBD and endogenous KLF1. In order to investigate the potential of competitive binding between GST-KLF DBD and endogenous KLF1, we performed chromatin immunoprecipitation (ChIP) in MEL cells expressing GST only, as well as those expressing GST-KLF1 DBD. Here we used an antibody against the N-terminus of KLF1 in order to detect endogenous KLF1 binding only in both cell types, as well as an antibody against GST, to detect the binding of GST-KLF1 DBD (Fig. 4A). The GST ChIP in GST only cells acted as a negative control, while IgG was used as an additional negative control in both cell types.

We were predominantly interested in investigating the effects of endogenous KLF1 binding upon the introduction of GST-KLF1 DBD, as well as the binding of GST-KLF1 DBD itself. Here we found that GST-KLF1 DBD is capable of binding to a number of KLF1 target sites, particularly the promoters of *Klf3* and *Klf8*, and that this binding appears to reduce the binding of endogenous KLF1 (Fig. 4B). We also found that GST-KLF1 DBD is capable of binding to some regions and not affect KLF1 binding, for instance at the *Lgals3* promoter, and that there are some regions where KLF1 is capable of binding, but GST-KLF1 DBD is not, for instance at the *Klf1* promoter. Taken together these results provide evidence for competition between transcription factors, but a complex and context dependent mechanism appears to be in operation.

## Discussion

We developed cell lines expressing GST fused upstream of the DNA binding domain of KLF1 (GST-KLF1 DBD), in order to probe the function of KLF1 in the erythroid/megakaryocyte switch. We discovered a pronounced phenotypic effect in these cells, providing further evidence for involvement of KLF1 in this process. Endogenous KLF1 levels are not altered in cells expressing GST-KLF1 DBD compared to cells expressing GST alone, suggesting that this phenotype is due to competition of GST-KLF1 DBD with endogenous KLF1. In support of this we found that the GST-KLF1 DBD protein is capable of binding to DNA, both *in vitro* and *in vivo*, and is capable of outcompeting endogenous KLF1 at particular KLF1 target sites, providing *in vivo* evidence of direct competition between transcription factors.

We initially found a decrease in the expression of a key KLF1 target gene, *Klf3*, in MEL cells overexpressing the GST-KLF1 DBD compared to GST only cells, at both the transcript and protein level. We also demonstrated a reduced erythroid phenotype and an increase in the megakaryocytic phenotype in MEL cells overexpressing this protein. These data support of the dual role of KLF1 in the erythroid/megakaryocyte switch, where KLF1 promotes erythroid differentiation, and represses megakaryocyte differentiation, and are indicative of a dominant negative affect of GST-KLF1 DBD on endogenous KLF1 in these lines.

While the MEL cells overexpressing GST-KLF1 DBD showed a marked reduction in erythroid features, the concomitant increase in megakaryocyte features was more subtle. Significant increases were seen only for CD41 cell surface antigen levels and for and *Gata2* and *Itga2b* expression at the mRNA level. *Gata1* expression was lower in the cells expressing GST-KLF1 DBD than cells expressing GST only, but this could be accounted for by the decreased erythroid phenotype in these cells as GATA1 is a key transcription factor in erythroid cells as well as megakaryocytes. It has previously been shown that MEL cells without KLF1 upregulate the expression of megakaryocyte specific genes such as *GpIX* and KLF1 is thought to block megakaryopoiesis via repression of *Fli1* ^22, 24, 25^. Interestingly, *Fli1* expression was not altered in our model. It is possible that the GST-KLF1 DBD does not have an effect at all KLF1 target genes for reasons discussed below. It is also plausible that the previously reported repression of *Fli1* by KLF1, was in fact modulated by KLF3. Our lab has previously shown that a number of non-erythroid genes are activated by KLF1, and attenuated by KLF3^18, 27^. It is conceivable that the level of downregulation of KLF3 in the cells expressing GST-KLF1 DBD is not enough to significantly affect *Fli1* expression in these cells.

Finally, we explored the potential mechanism underlying the observed dominant negative effects of GST-KLF1 DBD on endogenous KLF1. Steric hindrance, a concept established in prokaryotes, particularly at the *Escherichia coli lactose* (*lac*) operon^28^, is less well described in eukaryotic cells. In this example, the lac repressor protein (LacI) inhibits DNA binding of RNA polymerase, and subsequent gene activation^28^. This model can conceivably apply in eukaryotic cells to transcription factors with similar DNA binding specificities, whereby one factor preferentially binds competitively with factors possessing similar specificity. This mechanism would rely on a number of factors, including abundance of the various transcription factors involved, as well as their co-factors, and their relative DNA binding affinities. Many transcription factors are capable of binding similar DNA motifs, but which factors are actually bound at a given point in time *in vivo* is still an open research question. However, while there is much *in vitro* evidence of competition of transcription factors with similar sequence specificities, there are fewer examples where this has been observed *in vivo*. Here we investigated whether the GST-KLF1 DBD protein is capable of directly competing for binding sites at a number of known KLF1 targets.

In order to achieve this, we utilised the GST tag to perform parallel ChIP qPCR studies to examine the binding of both endogenous KLF1, and GST-KLF1 DBD in cells expressing either GST only, and those expressing GST-KLF1 DBD. In this experiment we found that GST-KLF1 DBD is capable of binding *in vivo* to some known KLF1 target sites, and that binding of endogenous KLF1 was significantly reduced at some, but not all, of these sites. The presence of KLF1 binding is markedly reduced at the *Klf3* and *Klf8* promoters, where GST-KLF1 DBD is found to bind. The expression of *Klf3* was found to be reduced in MEL cells expressing GST-KLF1 DBD compared to GST alone. This suggests that the binding of GST-KLF1 DBD is competitively inhibiting the binding of endogenous KLF1, and thereby acting as a dominant negative at some target sites. We have, however, found some sites, namely the *Klf1* promoter, where endogenous KLF1 is not significantly reduced, and the GST-KLF1 DBD fusion protein is unable to bind.

The observation that the GST-zinc finger DNA-Binding Domain is able to bind and compete with endogenous KLF1 at some targets and not others is at first surprising, but in fact fits with our prior observations studying KLF3 binding. We have previously performed experiments with a series of KLF3 mutants and found that a construct retaining only the minimal DNA-Binding Domain of KLF3 was able to bind some sites, but not others, *in vivo* ^29, 30^. We found that the functional domain of KLF3, that is the non-zinc finger N-terminal domain, is critical for the ability of wildtype KLF3 to locate and bind many of its target sites *in vivo*. This is likely due to interactions with various co-factors and other DNA-binding proteins that enable the proper targeting of this transcription factor to its target sites. This results with the GST-KLF1 DBD fusion protein investigated here, where the lack of the KLF1 N-terminal functional domain also shows effects on target gene specificity. It is likely that this protein is unable to interact with its normal cofactors, such as CBP/p300 and other SRC family members, and therefore, is unable to reach all of the CACCC sequences that wildtype KLF1 is able to bind to and regulate.

**Figure 5:**
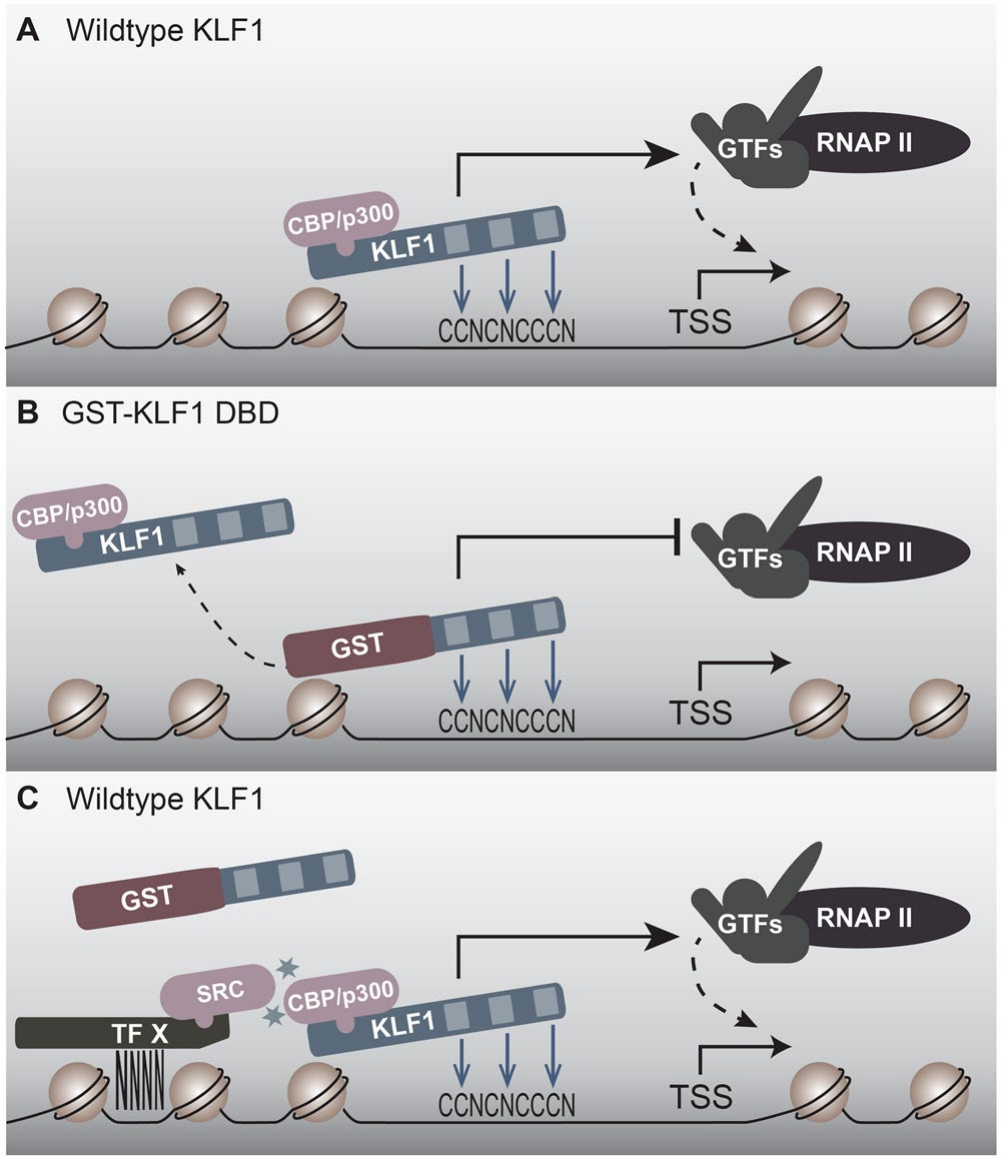
GST-KLF1 DBD can act as a dominant negative at some KLF1 target sites to displace endogenous KLF1 binding, thereby inhibiting DNA activation. (**A**) In the absence of GST-KLF1 DBD, endogenous KLF1 is able to bind and activate its target genes normally. (**B**) At some KLF1 target sites, for instance the *Klf3 1b* promoter, the GST-KLF1 DBD protein is able to out-compete endogenous KLF1, either by sheer amount of protein present, or a higher affinity for the site. This results in reduced activation of these target genes. (**C**) At some sites, GST is unable to bind due to it not being able to interact efficiently with other KLF1 partners and SRC family members. In this case, activation of KLF1 target genes in unaffected.

We therefore propose the following model for how the GST-KLF1 DBD protein is exerting its striking phenotypic effects in MEL cells (Fig. 5). Under normal conditions, KLF1, with the help of its co-factors CBP/p300 and other SRC family proteins, is able to locate and bind to all of its target sites *in vivo*, and thereby either activate, or in some instances repress, gene expression (Fig. 5A). However, at some KLF1 target sites, the interactions with co-factors and other DNA-binding proteins are less important, and it is at these sites that GST-KLF1 DBD is able to outcompete endogenous KLF1 and bind, thereby resulting in reduced activation of these target genes, such as *Klf3* and *Klf8* (Fig. 5B). However, at the sites where co-factors and other protein-protein interactions are more important for KLF1 binding, endogenous KLF1 is still capable of binding, and GST-KLF1 DBD is unable to bind, and therefore no altered effect on target gene expression is seen (Fig. 5C).

This mechanism can be more broadly applied to the potential competition between other transcription factors. KLF1 and KLF3, for instance, have similar DNA-binding preferences, and the two factors have been shown to bind *in vitro* to many of the same erythroid promoter CACCC-boxes^18, 20^, including that of the adult *β*-*globin* gene. KLF1, KLF3 and another related family member, KLF8, have been shown to be involved in a regulatory loop that plays a role in fine-tuning the expression of erythroid genes, including genes at the *β*-*globin* locus. That is, KLF1 is capable of activating a multitude of erythroid and non-erythroid genes, including *Klf3* and *Klf8*, and KLF3 plays a critical role in repressing a subset of these non-erythroid genes to maintain the correct expression program in erythroid cells^31^. It is not known, however, whether these factors are capable of binding competitively to the same target sites *in vivo*. Our results suggest that direct competition between these factors may be an important mechanism in gene regulation.

Future work will centre on investigating the properties of each of the KLF family members. This work will shed light on the importance of the functional domain in determining the DNA binding specificity of transcription factors as a whole.

## Methods

### Constructs and generation of stable lines

The vector pEF-IRES-puro5 (pEF1α) was provided by Dr Daniel Peet (University of Adelaide, Adelaide, Australia). GST was amplified from NpGEX2T, kindly provided by José Perdomo (School of Biochemistry, University of Sydney, Australia), using the following primers; 5′-GGTTTTCCATATGATTTTGGAGGATGGTCGCC-3′ and 5′-GGACTAGTTCATTTTGGAGGATGGTCGCC-3′, and cloned into pEF1α using the restriction enzymes NcoI and SpeI. The KLF1 DBD domain (261–376) was amplified from murine spleen cDNA using the following primers; 5′-CCGGAATTCGGGGCCACTGCGATCGCC-3′ and 5′-CGCGGATCCTCAGAGGTGACGCTTCATGTG-3′, and cloned into pEF1α using the restriction enzymes NcoI and SpeI.

Mouse erythroleukaemia (MEL) cells were maintained in RPMI1640 (Life Technologies) supplemented with 10% fetal calf serum (FCS; Life Technologies) and 1% penicillin, streptomycin and L-glutamine (PSG, Life Technologies). Stably expressing cells were created using the Neon Transfection System (Life Technologies). Cells (105) were resuspended in nucleofection buffer T (Neon Transfection Kit, Life Technologies) and given three pulses of 1,450 V for 20 milliseconds. Cells were then cultured for 48–72 h in RPMI1640 supplemented with 10% FCS before selection. After this time MEL cells were selected with 0.5 µg/mL puromycin for 5 days before single cell sorting using the FACS Aria II (BD Bioscience). Expression of desired constructs was analysed by Western blot.

For induction of MEL cells, cells were cultured for 72 hours in RPMI1640 supplemented with 10% FCS, 1% PSG, and 2% DMSO, as previously described^26^.

### Cytoplasmic and nuclear extracts, and Western blots

20 µg of cytoplasmic or nuclear fractions were obtained, and Western blots were performed by standard methods. Briefly, extracts were separated using 10% Bis-Tris NuPAGE gels (Thermo Fisher Scientific) and were electrotransferred to nitrocellulose membrane, which was blocked with 4% skim milk in 50 mM Tris-HCl (pH 7.5), 150 mM NaCl, 0.05% Tween-20 (TBST). GST and KLF3 proteins were probed for 1 hour in TBST using 0.2 µg/mL anti-GST antibody (1:5,000 dilution - ab19256, Abcam) or 0.2 µg/mL anti-KLF3 (1:5,000 dilution - PA5–18030, Thermo Fisher Scientific), respectively. KLF1 proteins were probed overnight in 4% skim milk in TBST using a KLF1 antibody that recognises both endogenous mouse KLF1 and GST-KLF1 DBD (1:1,000 dilution, antibody generation reported previously)^20^. The Immobilon Western Chemiluminescent HRP Substrate System (Millipore Corporation) was used for detection. Membranes were subsequently stripped using 0.2 M NaOH for 10 minutes and were probed with anti-β-actin (1:30,000 dilution - A1978, Sigma) as a loading control.

### Electrophoretic mobility shift assay (EMSA)

EMSAs were performed as previously described^20^. The radiolabelled probe containing the KLF1 consensus binding site comprises sense and antisense oligonucleotides for the sequence 5′-TAGAGCCACACCCTGGTAAG-3′, as described^20^. 2 µl of the same antibodies used for Western blot analysis were used in the supershift lanes.

### Densitometry

Densitometry of relative band intensities was performed using ImageJ Gel Analysis tool^32, 33^. Briefly, ImageJ was used to select and determine the background-subtracted density of the bands for KLF3 in both the EMSAs and western blots. In the EMSAs, KLF3 was normalised to SP1 as a loading control, and in the Western blot analysis KLF3 and KLF1/GST-KLF1 DBD bands were normalised to the loading control Actin.

### Flow Cytometry

Flow cytometry was performed using the LSRFortessa Flow Cytometry (BD Bioscience), and the data analysed using FlowJo v7.6.5 (TreeStar) Software. The mouse antibodies TER119 (BD Bioscience), CD71 (clone RI7217, Biolegend), CD41 (clone MWReg30, BD Bioscience) and CD42a (clone Xia.B4, Emfret Analytics) were used at the concentrations recommended by the manufacturers.

### cDNA synthesis and real time RT-PCR (qPCR)

Total RNA was extracted using the RNeasy Mini Kit (Qiagen) as per the manufacturer’s instructions. The DNA-free kit (Ambion) was used to eliminate genomic DNA contamination. 5 µg of RNA was converted to cDNA with the SuperScript VILO cDNA synthesis kit (Invitrogen) for use in real time qPCR.

Paired primers were designed using the PrimerExpress software (Version 2.0) (Applied Biosystems) to cross exon-exon boundaries, where possible, in order to prevent the amplification of potential contaminating genomic DNA. Primer specificity was checked using the Basic Local Alignment Search Tool (BLAST; http://www.ncbi.nlm.nih.gov/BLAST)^34^. The oligonucleotide sequences for murine primers are as follows: *18S*, 5′-CACGGCCGGTACAGTGAAAC-3′ and 5′-TTGGTCGCTCGCTCCTCT-3′; *Klf1*, 5′-AGACTGTCTTACCCTCCATCAGTACA-3′ and 5′-CCGCCACCACTTGAGGAA-3′; *Klf3*, 5′-GAAATGTCACCCCCTTTAATGAAC-3′ and 5′-ACCATCCTTCCGTCATCGTG-3′; *Hba*-*x*, 5′-ATGCGGTTAAGAGCATCGAC-3′ and 5′-CAACTTCAAGCTCCTGTCCC-3′; *Hba*-*a1*, 5′-CTCTGGGGAAGACAAAAGCA-3′ and 5′-ACTTTGATGTAAGCCACGGC-3′; *Hbb*-*y*, 5′-GGCCTGTGGAGTAAGGTCAA-3′ and 5′-TTTGGGAACTTGTCCTCTGC-3′; *Hbb*-*bh1*, 5′-AATCACCAGCTTCTGCCAGGC-3′ and 5′-TGAGCAAAGGTCTCCTTGAG-3′; *Hbb*-*b1*, 5′-CACTGTGACAAGCTGCATGT-3′ and 5′-CTGGCTCACAAGTACCACTA-3′; *Gata2*, 5′-AGAACCGGCCGCTCATC-3′ and 5′-TCGTCTGACAATTTGCACAACAG-3′; *Runx1*, 5′-CCCGCCTTCAGGAGAGGTGC-3′ and 5′-GGAAGGCGGCGTGAAGCG-3′; *Fli1*, 5′-ACCAGCCAGTGAGAGTCAAT-3′ and 5′-CCGTTCTTCTCATCCATGTA-3′; *GPiX*, 5′-TACCAGCCCACAAAAGGTGT-3′ and 5′-GGGCAAGCCTGAGTATCTGT-3′; *Itga2b*, 5′-CCCTGTCGCGCCAACACCAT-3′ and 5′-CAGCCTGGGTCACCGCCAAG-3′.

qPCR runs were performed in triplicate with Power SYBR green PCR Master Mix and using the 7500 Fast Real time PCR system (Applied Biosystems) as previously described^35^. Approximately 10 ng of cDNA and a final concentration of 2 µM of primer were used per reaction.

### Chromatin immunoprecipitation (ChIP)

ChIP was performed based on the protocol by Schmidt *et al*.^36^, with slight modifications. Briefly, 1.5 × 10^8^ cells were crosslinked using formaldehyde for 20 minutes. Each pellet was used for three IPs, with the following antibodies each at 15 µg, Goat IgG (sc-2028, Santa Cruise), GST (ab-19256, Abcam) and KLF1 (PA5-18031, Thermo Fisher Scientific). Real-time PCR quantification of chromatin pull-down was performed as described above and amounts were normalised to the level of input material prior to immunoprecipitation. In order to equate for subtle differences across samples, each antibody for each cell type was normalised to the most negative region. The primer sequences are as follows; *Klf3* Promoter, 5′-CTGGGTGTGGGCAGAATCTT-3′ and 5′-AGTTGGACTCGCCCTGGC-3′; *Klf8* Intron, 5′-CCAGCTCGTGCACACTGAA-3′ and 5′-TTCCACTCCTGATGTTAAGGCTTC-3′; *Klf1* Promoter, 5′-CACAACAGAGCAATTCAAAGCTAAA-3′ and 5′-CTCAAGGAGGAACAGAGCTATGG-3′; *Lgals3* Promoter, 5′-TGGAAAAACACCCGTGCCTCTGA-3′ and 5′-GAGTCATCTGGGCGTAGGCACTG-3′; *Klf8* +33 kb, 5′-AACCTGGGTGCCTCCTTGTA-3′ and 5′-AAAGCACTAAAGTCAAAGGCATGA-3′.

## Electronic supplementary material

Supplementary Info
