# Supplementary material for: Direct competition between DNA binding factors highlights the role of Krüppel-like Factor 1 in the erythroid/megakaryocyte switch
Source: Sci Rep. 2017 Jun 9;7:3137. doi: 10.1038/s41598-017-03289-5 (PMC5466599; doi:10.1038/s41598-017-03289-5)
Supplement: Supplementary file 1 — Supplementary Info [file 41598_2017_3289_MOESM1_ESM.pdf]

Direct competition between DNA binding factors highlights the role of Krüppel-like Factor 1 in the erythroid/megakaryocyte switch

Laura J. Norton[§1], Samantha Hallal[§2], Elizabeth S. Stout[1], Alister P. W. Funnell[1,2], Richard C. M. Pearson[1,2], Merlin Crossley[1,2] and Kate G. R. Quinlan*[1]

[§]Equal first authors

*Corresponding author

From [1]School of Biotechnology and Biomolecular Sciences, University of New South Wales, NSW, 2052, Australia., and [2]School of Molecular Bioscience, University of Sydney, NSW, 2006, Australia.

To whom correspondence should be addressed: School of Biotechnology and Biomolecular Sciences, University of New South Wales, Sydney, New South Wales 2052, Australia. Tel.: 61-2-9385-8740; E-mail: kate.quinlan@unsw.edu.au.

Supplementary Results

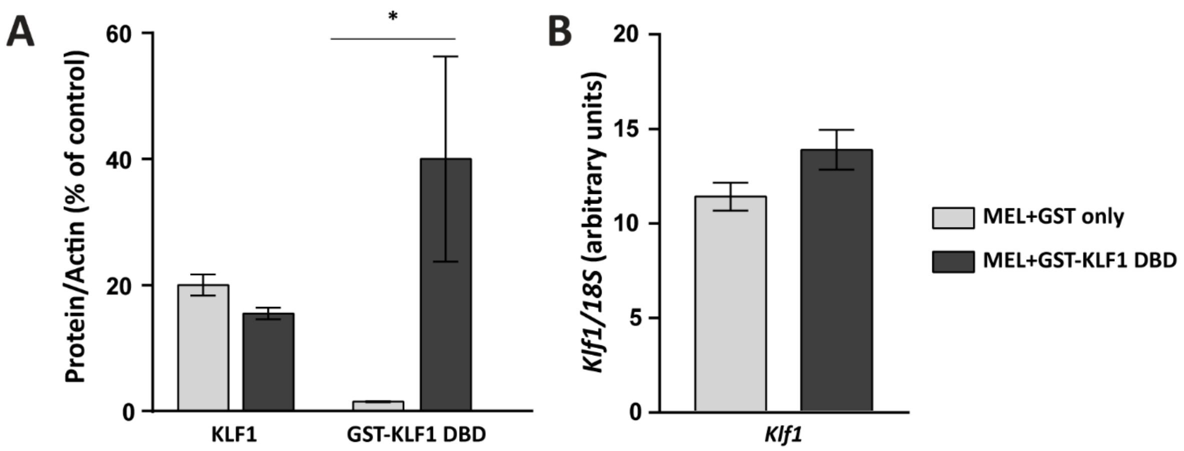

Figure S1. KLF1 levels are unchanged in MEL cells expressing GST-KLF1 DBD. (A) Densitometry analysis of Western blots performed in MEL+GST only compared to MEL+GST-KLF1 DBD expressing clones, measured using ImageJ analysis software. Intensity of KLF1 and GST-KLF1 DBD bands were normalised to the Actin loading control. **(B)** Total *Klf1* transcript levels were analysed by qPCR in MEL+GST only compared to MEL+GST-KLF1 DBD expressing clones. Error bars represent the standard error of the mean. n = 4 for each construct, p values indicate the difference between the means, *, $p < 0.05$ (paired Student's two-tailed t test).

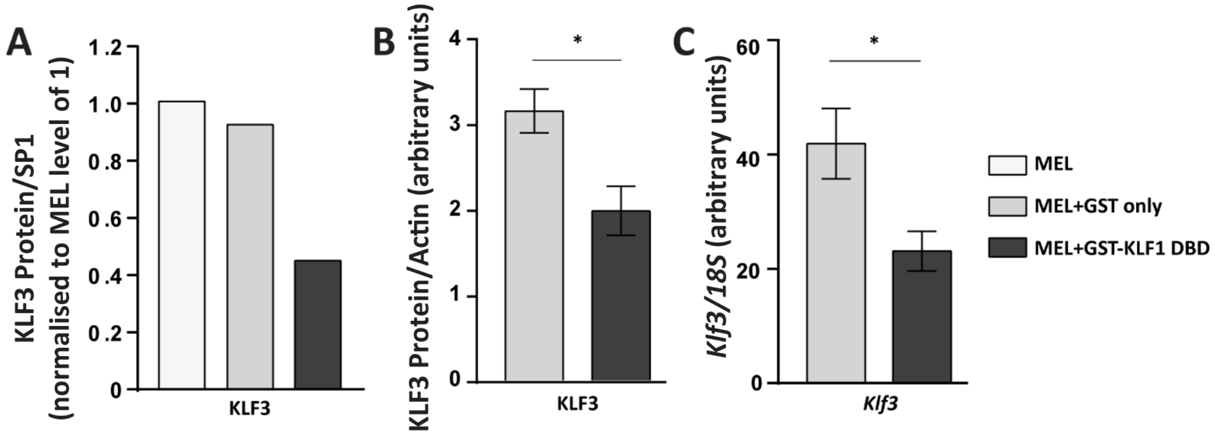

Figure S2. KLF3 levels are decreased in MEL cells expressing GST-KLF1 DBD. (A) Densitometry analysis of EMSAs performed in MEL+GST compared to MEL+GST-KLF1 DBD expressing clones. **(B)** Densitometry analysis of Western blots performed in MEL+GST only compared to MEL+GST-KLF1 DBD expressing clones, measured using ImageJ analysis software. Intensity of KLF3 bands were normalised to the Actin loading control. **(C)** Total *Klf3* transcript levels were analysed by qPCR in MEL+GST only compared to MEL+GST-KLF1 DBD expressing clones. Error bars represent the standard error of the mean. n = 4 for each construct, p values indicate the difference between the means, *, $p < 0.05$ (paired Student's two-tailed t test).

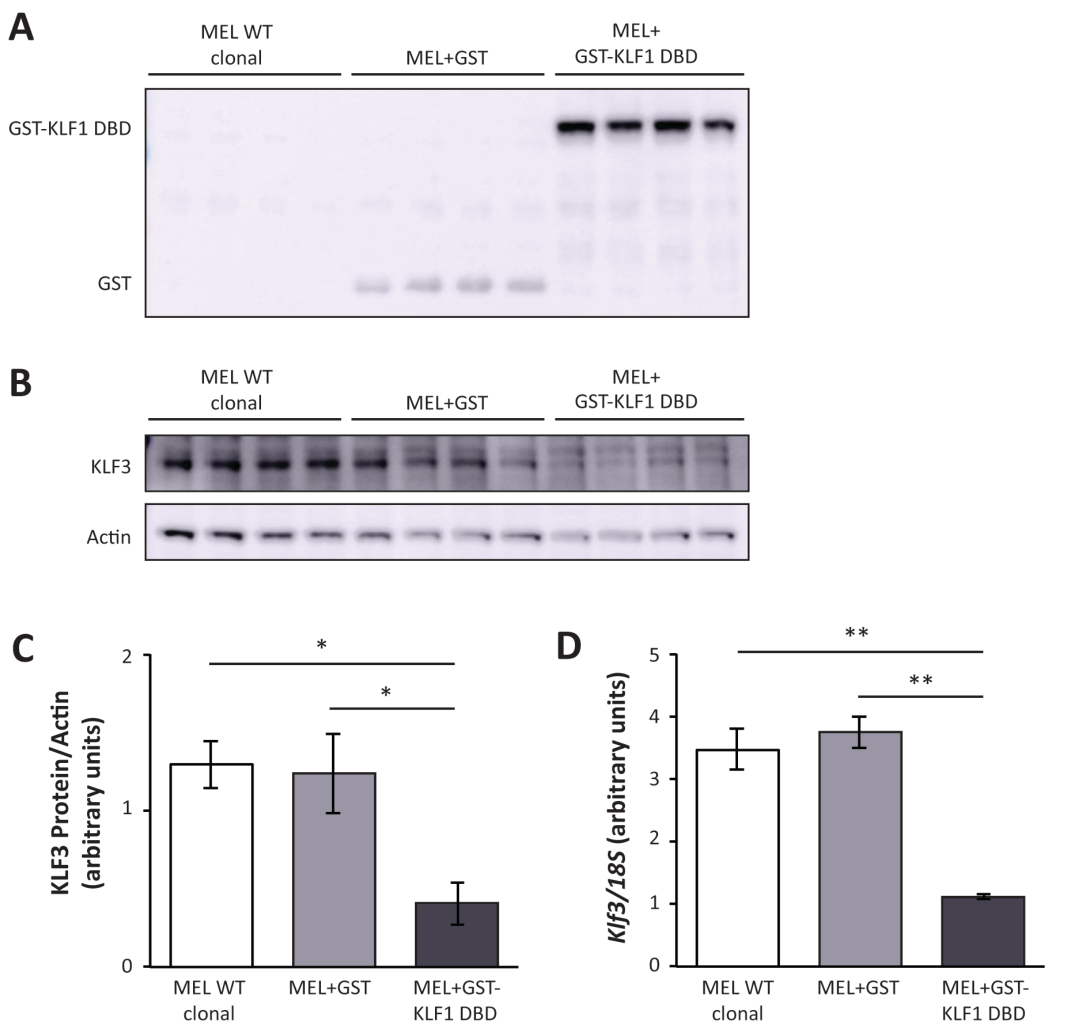

Figure S3. KLF3 levels are decreased in MEL cells expressing GST-KLF1 DBD compared to GST alone and clonal populations of parental MEL cells. (A) Western blots from MEL lines using an antibody that recognises GST, demonstrating protein expression of GST and GST-KLF1 DBD for each clone, in comparison to concurrently generated clonal parental MEL cell lines. **(B)** Western blots from MEL clones with an antibody that recognises KLF3, indicating reduced expression of endogenous KLF3 in cells overexpressing GST-KLF1 DBD compared to GST only clones and compared to concurrently generated clonal parental MEL cell lines. β-Actin is presented as a loading control. n = 4 for each construct. **(C)** Densitometry analysis of Western blots performed in MEL+GST only, MEL+GST-KLF1 DBD expressing clones and concurrently generated clonal parental MEL cells (from (B)), measured using ImageJ analysis software. Intensity of KLF3 bands were normalised to the Actin loading control. **(D)** Total *Klf3* transcript levels were analysed by qPCR in MEL+GST only compared to MEL+GST-KLF1 DBD expressing clones and concurrently generated clonal parental MEL cells. Error bars represent the standard error of the mean. n = 4 for each construct, p values indicate the difference between the means, *, $p < 0.05$; **, p , <0.001 (paired Student's two-tailed *t* test).
